# What a Difference a Day Makes: Examining the Lag Patterns of PM_2.5_ Constituents

**DOI:** 10.1289/ehp.120-a321b

**Published:** 2012-08-01

**Authors:** Tanya Tillett

**Affiliations:** Tanya Tillett, MA, of Durham, NC, is a staff writer/editor for *EHP*. She has been on the *EHP* staff since 2000 and has represented the journal at national and international conferences.

Exposure to fine particulate matter (PM_2.5_) is associated with a number of adverse health conditions, including cardiovascular and respiratory ailments, but less is known about associations with its specific chemical constituents. Past studies on the short-term effects of PM_2.5_ constituents have relied on data from monitoring networks that sample every third or sixth day. Now researchers report their findings on the daily lag patterns between exposure to individual PM_2.5_ constituents and hospital admissions for various conditions [*EHP* 120(8):1094–1099; Kim et al.].

PM_2.5_ is a complex mixture of water droplets and solid particles of carbon, nitrate, sulfate, metals, trace elements, and other constituents. In this study, researchers used information provided by the Denver Aerosol Sources and Health (DASH) study, which measured PM_2.5_ constituents daily for five years (2003–2007) in the Colorado capital. They focused on four of the main constituents of PM_2.5_—elemental carbon (EC), organic carbon (OC), sulfate, and nitrate.

The researchers obtained data on discharge diagnoses following nonelective hospital admissions (e.g., emergency, urgent, or trauma care) in the five-county Denver metropolitan area. They categorized cardiovascular diagnoses as ischemic heart disease, congestive heart failure, or cerebrovascular disease, and respiratory diagnoses were categorized as chronic obstructive pulmonary disease, asthma, or pneumonia. The investigators analyzed lag patterns for exposure to each PM_2.5_ constituent and hospital admissions for the six cardiovascular and respiratory illnesses on the day of admission and each of 14 days prior.

On average there were 236 nonelective hospital admissions per day; 19% of these patients had a discharge diagnosis of cardiovascular disease, and 16% had a discharge diagnosis of respiratory disease. Cardiovascular hospital admissions were most strongly associated with a lag of 0–1 day, with the pattern most dominant for EC and OC, and for ischemic heart disease. Respiratory hospital admissions were most strongly associated with a longer lag of 2–5 days, with the pattern again most dominant for EC and OC, and with asthma showing the strongest associations. Sulfate and nitrate showed less association with any diagnosis at any lag. Lag patterns were consistent after adjusting for exposure to the gaseous copollutants carbon monoxide, sulfur dioxide, nitrogen dioxide, and ozone.

The study is limited by its reliance on only one residential monitoring site for ambient PM_2.5_ and its chemical constituents, but the authors write that the strong correlations between the data obtained from that site and from a nearby federal monitoring site support the validity of the study design. The authors plan future studies to explore whether specific sources of PM_2.5_ exhibit similar time lag effects.

